# Telomere Length Associates With Symptom Severity After Mild Traumatic Brain Injury in Older Adults

**DOI:** 10.1089/neur.2023.0012

**Published:** 2023-05-23

**Authors:** Sarah R. Martha, Ernesto J. Tolentino, Andrew A. Bugajski, Hilaire J. Thompson

**Affiliations:** ^1^ Biobehavioral Nursing Science Department, College of Nursing, University of Illinois at Chicago, Chicago, Illinois, USA University of Washington, Seattle, Washington, USA.; ^2^ Office of Nursing Research, University of Washington, Seattle, Washington, USA.; ^3^ Department of Research and Sponsored Studies, Lakeland Regional Health Medical Center, Lakeland, Florida, USA.; ^4^ Biobehavioral Nursing and Health Informatics Department, School of Nursing, University of Washington, Seattle, Washington, USA.; ^5^ Harborview Injury Prevention and Research Center, University of Washington, Seattle, Washington, USA.

**Keywords:** aged, head injury, Rivermead Post-Concussion Symptoms Questionnaire

## Abstract

The objectives were to compare differences in telomere length (TL) among younger (21–54 years) and older adults (≥55) with mild traumatic brain injury (mTBI) to non-injured controls and to examine the association between TL and the severity of post-concussive symptoms over time. We performed a quantitative polymerase chain reaction to determine the TL (Kb/genome) of peripheral blood mononuclear cell samples (day 0, 3 months, and 6 months) from 31 subjects. The Rivermead Post-Concussion Symptoms Questionnaire was used to assess symptoms. Group-by-time comparisons of TL and symptom severity were evaluated with repeated-measures analysis of variance. Multiple linear regression examined the relationship between TL, group (mTBI and non-injured controls), and symptom severity total and subscale scores. Significant aging-related differences in TL were found within mTBI groups by time (day 0, 3 months, and 6 months; *p* = 0.025). Older adults with mTBI experienced significant worsening of changes in total symptom severity scores over time (day 0, 3 months, and 6 months; *p* = 0.016). Shorter TLs were associated with higher total symptom burden among each of the four groups at day 0 (baseline; *p* = 0.035) and 3 months (*p* = 0.038). Shorter TL was also associated with higher cognitive symptom burden among the four groups at day 0 (*p* = 0.008) and 3 months (*p* = 0.008). Shorter TL was associated with higher post-injury symptom burden to 3 months in both older and younger persons with mTBI. Large-scale, longitudinal studies of factors associated with TL may be useful to delineate the mechanistic underpinnings of higher symptom burden in adults with mTBI.

## Introduction

Globally, the annual incidence of traumatic brain injury (TBI) affects an estimated 27 to 69 million persons.^1,2^ Mild TBI (mTBI) accounts for the majority (>75%) of brain injuries. Of those with mTBI, older adults (>65 years of age) are over-represented, accounting for at least 22% of those injured, despite being only 13% of the population.^3,4^ Older adults with mTBI experience higher rates of morbidity and mortality, along with worsening symptoms, compared to those of younger patients with mTBI.^5,6^ Although termed mild, the initial insult disrupts normal brain function in response to mechanical force. The different types of mechanical forces involved in TBI include acceleration/deceleration, rotational forces, and blast and blunt injuries.^7^ These forces directly damage neurons, glia cells, and vasculature in a focal, multi-focal, or diffuse pattern and activate cellular and inflammatory pathways.^7^ After a mild TBI, the resulting cellular processes involve damaged DNA, including telomeres.^8,9^

Telomeres are nucleoprotein structures that represent the ends of genomic chromosomes. They are hexamer repeats of the nucleotide sequence [TTAGGG] and play a critical role in maintaining structural stability and protecting DNA against genetic information loss and damage.^10^ Inherent to cell division, telomeres degrade over time and the number of hexamer repeats is reduced after each cycle of replication.^9^ Under normal conditions, each cell division results in the telomere length (TL) shortening by ∼30–200 base pairs.^11^ The shorter the TL, the closer the cell is to senescence and/or apoptosis.^12^ Shortening of telomeres can result in decreased levels of chromosomal stability and has been shown to be associated with the aging process as well as neurodegenerative diseases.^10,13,14^ Any disruption from normal brain function, either attributable to mechanical injury (e.g., mTBI) or pathological changes (e.g., normal aging) in neurons, can result in the development or progression of neurodegeneration that may impact quality of life, including higher symptom severity.^15^ There is limited research examining the association of TL after mTBI and symptom burden in aging adults.

Recent literature suggests that telomere shortening may be accelerated by TBI. Most studies using TL as a marker for outcomes after mTBI are conducted in adolescent and younger adult humans and rodents, and without longitudinal sampling. Machan and colleagues found no significant associations between TL and history of concussion, age, or sport contact type in a cohort of varsity athletes (*n* = 183; 16–27 years of age) across multiple sports.^16^ Symons and colleagues found that Australian football players (*n* = 95; mean, 23 years) had shorter salivary TL compared to control athletes (*n* = 49; mean, 23 years), but history of concussion was not associated with salivary TL.^17^ In a juvenile concussive rodent model, shorter TL is associated with history of mTBI, and rats with shorter TL performed worse on a battery of behavioral tests (e.g., cognition, memory, anxiety-like, and depressive-like symptoms) in the acute/subacute period after concussion.^18^

These studies suggest that TL may be a biomarker for outcomes after mTBI. In addition, shortened TL is associated with aging and may represent a biomarker of neurological health.^19^ However, currently little is known about longitudinal changes in TL after mTBI in older adults and whether this biomarker may be useful in predicting and understanding mechanisms underlying symptom severity. The purpose of this study was to 1) compare differences in TL among younger (21–54 years) and older adults (≥55) with mTBI as well as non-injured controls and 2) examine the association between TL and severity of post-concussive symptoms over time.

## Methods

### Study design

The present investigation is a secondary analysis of peripheral blood mononuclear cell (PBMC) samples taken from a prospective cohort study. The parent study assessed adults with mTBI and non-injured control groups to explore relationships among aging, TBI, immunological biomarkers and functional outcomes. This investigation uses data and biospecimens from four groups in that study: 1) older (≥55 years) and 2) younger (21–54 years) participants who experienced mTBI; and non-injured 3) older (≥55 years) and 4) younger (21–54 years) adult participants. The cut-off point for younger and older adults was selected based on field trauma triage guidelines of the American College of Surgeons' Committee on Trauma and the Centers for Disease Control and Prevention (CDC), which delineate older adults as ≥55 years.^20^

### Parent study setting and participants

Information on research site, eligibility and recruitment, and procedures for the parent study was previously published.^21^ In brief, older and younger adult TBI study participants were recruited prospectively from a level 1 trauma center in Seattle, Washington. The non-injured older and younger adults were recruited from the surrounding metropolitan area.

To be eligible for the parent study, cases had to be ≥55 years of age for the older adult group or 21–54 years of age for the younger adult group, diagnosed with mTBI (by CDC criteria)^22^ within the past 24 h. Exclusion criteria included persons with cervical spine trauma; previous stroke or TBI within the past year; diagnosis of dementia; and moderate injury to other body regions (Abbreviated Injury Scale >2).^23^

Non-injured control participants were candidates for inclusion if they were ≥55 years of age for the older adult group or 21–54 years of age for the younger adult group and reported an ability to perform independent activities of daily living without assistance. Participants were excluded if they had a previous stroke or TBI or diagnosis of dementia. The University of Washington's (UW) Institutional Review Board approved the study, and participants provided written informed consent.

### Parent study procedures

Investigators collected blood samples from all participants within 24 h of injury or at the time of enrollment for controls, in addition to months 1 and 6 for all participants. In brief, ∼12 mL of blood was collected from each participant and split into two ethylenediaminetetraacetic acid–anticoagulated blood tubes (Becton Dickinson, Franklin Lakes, NJ) for PBMC isolation. All samples were initially processed, aliquoted in 250 μL, and stored at −70°C or in liquid nitrogen within 4 h of collection. Briefly, after 10 min of centrifugation at 1000*g*, the buffy coat layer was then pipetted off the surface of the red blood cell layer with 15 mL in phosphate-buffered saline (PBS) solution and gently mixed. This solution was then carefully layered on top a pre-measured 10-mL solution of Ficoll-Paque (GE HealthCare, Chicago, IL) contained in a 50-mL tube. The sample was centrifuged for 30 min at 700*g*, at 15°C. The mononuclear cell layer was then harvested and placed in a new 15-mL tube and brought up to 10 mL with sterile PBS solution. After centrifugation at 1000 rpm for 5 min, the cell pellet was then resuspended in 5-mL of PBS. To determine PBMC concentration, a 20-μL portion was counted with a hemocytometer. Cells were pelleted by centrifugation at 1000 rpm for 5 min and adjusted to a concentration of 10^7^ cells/mL with complete Freezing Media (fetal calf serum [FCS] containing 10% dimethyl sulfoxide) in Nunc (catalog #264300; ThermoFisherScientific, Waltham, MA) storage vials (1 mL per vial). Tubes were then placed into a room-temperature Slo-Cooler box and placed in a −70°C freezer overnight. The next day, these tubes were transferred into liquid nitrogen for long-term storage.

Demographic data, injury data (type, location, mechanism, and severity for mTBI participants only), and comorbid conditions were collected at enrollment from the medical record. For control participants, demographic data and comorbid conditions were collected by interview at enrollment.

The Rivermead Post-Concussion Symptoms Questionnaire (RPQ) reflects the presence and severity of post-concussive symptoms and was collected at enrollment (day 0) and 3 and 6 months.^24,25^ The RPQ has a total of 16 questions, and each item is rated 0–4 comparing the symptoms (e.g., headaches, feelings of dizziness, nausea, fatigue, and poor concentration) to pre-injury (0 = not experienced at all; 1 = no more of a problem; 2 = a mild problem; 3 = a moderate problem; and 4 = a severe problem). Total score ranges from 0 to 64, where higher scores reflect greater severity of post-concussive symptoms.^26^ We utilized the three-factor symptom subscale (cognition, emotion, and somatic) of the RPQ.^25^ The cognition symptom cluster includes items of forgetfulness, concentration, and longer to think. The emotion symptom cluster includes symptoms of irritability, depression, frustration, and restlessness. The somatic symptom cluster is comprised of fatigue, headache, dizziness, nausea, sleep disturbances, blurred vision, and photophobia.^25^

### Sampling

Participants (*N* = 31; *n* = 11 older adults with mTBI; *n* = 7 younger adults with mTBI; *n* = 7 non-injured older controls; and *n* = 6 non-injured younger controls) in the present study were a randomly selected subset of participants in the parent study. Frozen PBMC samples from three time points (day 0, 3 months, and 6 months), from the four groups of participants, were processed in the UW School of Nursing Biobehavioral Laboratory for relative TL analyses.

### Protocol for relative-length telomere determination by reverse-transcriptase polymerase chain reaction methods

#### Isolation/extraction of DNA from peripheral blood mononuclear cells

DNA isolation was performed by using the Gene JET Whole Blood Genomic DNA purification protocol (catalog #K0781; ThermoFisherScientific). Thawed PBMC cells were washed twice with 10 mL of cold PBS, to remove FCS freezing media, then centrifugation at 1300 rpm for 5 min. The pellet was resuspended in 200 μL of PBS after the final wash. To the pellet, 20 μL of proteinase K was added and incubated at 56°C in a shaker bath, to lyse the cells. Then, 200 μL of ethanol was added to the mixture in an extraction column supplied by the kit manufacturer. After a series of washing steps, a 400-μL elution buffer was added to the spin column to isolate DNA in solution. The DNA sample was then frozen at −70°C until the telomere assay.

#### Quantitation of DNA

DNA was quantified using the NanoDrop (ND-1000; ThermoFisherScientific) spectrophotometer, using 2 μL of sample, and measured at 280 nm. Values are reported in ng/mL.

#### Telomere polymerase chain reaction

Relative human telomere quantitation was performed on DNA samples (ScienCell qPCR RHTLQ; Catalog #8908; ScienCell Research Laboratories, Inc., Carlsbad, CA). In brief, each DNA sample was treated with two quantitative polymerase chain reaction reactions, one with telomere primer stock solution, and one with single-copy reference (SCR) primer stock solution (SCR is the single-copy reference primer set), along with TaqGreen master mix (supplied). Samples were run in triplicate. After a brief mixing and centrifugation step, the polymerase chain reaction thermal cycler (Applied Biosystems Quant3 Studio; Applied Biosystems, Foster City, CA) performed the following cycle conditions: denaturation at 95°C for 10 min, followed by 32 cycles of 95°C for 20 sec (denaturation), 52°C for 20 sec (annealing), and 72°C for 45 sec (extension). Data acquisition was obtained automatically and is expressed as both telomere and single-copy delta Cq. To determine relative TL between individual DNA samples, the ΔΔCq formula was used: ΔΔCq = ΔCq (TEL) – ΔCq (SCR).^27^

### Statistical analysis

SPSS software (version 28.0; IBM, Armonk, NY) was utilized for statistical analyses. Means, standard deviations, frequency distribution, and percentages were used to characterize older and younger adults with mTBI and non-injured older and younger controls. On initial examination, investigators found that distributions of TL were unevenly distributed and formed two distinct groupings, which were then dichotomized into “short” and “long” TL. Variables describing older and younger mTBI groups produced heteroscedasticity and collinearity errors that could not be reconciled and was therefore collapsed into the mTBI group and the non-injured control group in the regression model.

We used repeated-measures analysis of variance (RM-ANOVA) to examine TL changes from day 0 to 3 and 6 months by group (mTBI and non-injured controls). We examined TL for differences both within and among each group. We compared RPQ symptom severity total and subscale (cognition, emotion, and somatic) scores both at each time point (day 0, 3 months, and 6 months) and by group (mTBI or non-injured controls). Symptom severity scores were examined for differences within and between groups. Student Newman-Keuls (SNK) *post hoc* comparisons were carried out, when appropriate. We used multiple linear regression to examine the relationship between TL, group (mTBI and non-injured controls), and RPQ symptom severity scores (total and subscale [cognitive, emotion, and somatic]). Statistical significance was set as *p* < 0.05. For all graphs, means ± standard error are displayed.

## Results

### Participant and symptom characteristics

Thirty-one total participants were included in the present study; demographic and injury-related characteristics are shown in Table 1. Older participants with mTBI (*n* = 11) and older controls (*n* = 7) were both, on average, 77 years of age, whereas younger adults with mTBI (*n* = 7) averaged 32 years of age; younger controls averaged 30 years of age. All four groups were primarily white and mostly male, except that older non-injured controls were mostly female (see Table 1). The most common comorbid conditions in both older groups were cardiac arrhythmias, hypertension, thyroid disorder, and diabetes (Table 1). The primary mechanism of injury for older adults with mTBI in the sample was fall (72.7%), whereas for younger participants with mTBI the mechanism was motor vehicle crash (42.9%), followed by struck by or against (28.6%) and bicycle hit by vehicle (28.6%). The majority of older and younger adults with mTBI received pre-hospital care (Table 1). There was a significant difference between older and younger adults related to a positive computed tomography (CT) scan on admission (45.5% and 28.6%, respectively). With regard to symptoms, average RPQ scores at day 0, 3 months, and 6 months in older adults post-mTBI were 25.1 ± 8.1, 18.0 ± 7.1, and 17.1 ± 3.6, respectively, and younger mTBI participants exhibited scores of 21.4 ± 9.1, 14.6 ± 4.8, and 12.6 ± 3.5, respectively.

**Table 1.** Participant and Outcome Characteristics

Characteristic	Older adults with mild TBI (*n* = 11)	Younger adults with mild TBI (*n* = 7)	Non-injured older controls (*n* = 7)	Non-injured younger controls (*n* = 6)
Male sex (%)	54.5	57.1	14.3	83.3
Age, years	77.0 ± 10.1	32.0 ± 9.0	77.0 ± 9.1	30.0 ± 7.2
Hispanic ethnicity (%)	0	0	0	0
Race Black/African American White Asian	— 100% —	28.6% 71.4% —	— 85.7% 14.3%	16.7 66.7 16.7
Common chronic conditions (%) Cardiac arrhythmias Hypertension Thyroid disorder Diabetes Fluid and electrolyte disturbances Congestive heart failure Atherosclerosis Depression No comorbid conditions	45.5 27.3 27.3 18.2 9.1 9.1 9.1 9.1 —	— — — — — — — — 100	14.3 57.1 14.3 28.6 — — — — 28.6	— — — — — — — 50 50
Mechanism of injury (%) Fall MVC-driver Struck by or against Bicycle hit by vehicle Unknown	72.7 9.1 — 9.1 9.1	— 42.9 28.6 28.6 —		
Pre-hospital care received (%)	81.8	85.7		
Positive CT scan (%) Yes	45.5	28.6		
Hospital length of stay (days)	2.0 ± 2.3	0.5 ± 0.5		
Glasgow Coma Scale Admission to hospital	14.9 ± 0.3	14.4 ± 1.5		
Rivermead Post-concussion Symptoms Questionnaire Day 0 3 months 6 months	25.1 ± 8.1 18.0 ± 7.1 17.1 ± 3.6	21.4 ± 9.1 14.6 ± 4.8 12.6 ± 3.5	10.4 ± 4.5 7.3 ± 6.0 9.9 ± 7.5	10.5 ± 2.8 10.3 ± 7.0 9.8 ± 4.5

Data are reported as mean ± SEM unless indicated.

SEM, standard error of the mean; MVC, motor vehicle crash; CT, computed tomography.

### Telomere length by group

**FIG. 1.**
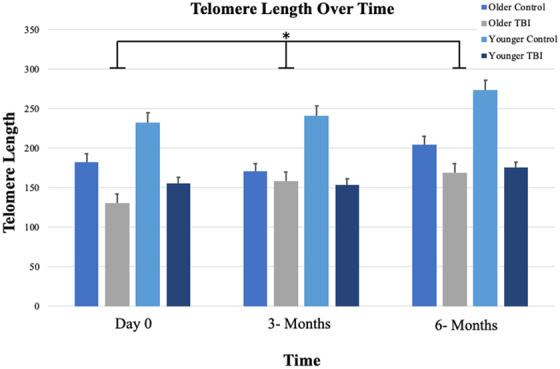
Aging-related differences were observed within mild TBI groups × time in telomere length (TL). TL of the four groups increased over time (*p* = 0.023), but these differences were not significant (*p* = 0.089). Line graph displays mean ± standard error of the mean. *Indicates a significant main effect of time. TBI, traumatic brain injury.

We evaluated comparisons between mTBI participants and their non-injured controls across three time points (day 0, 3 months, and 6 months) in TL (Fig. 1). Older TBI and older control groups had shortened TL compared to younger TBI and younger control groups, respectively. RM-ANOVA identified significant aging-related difference within mTBI groups by time in TL, but differences were not significant between older and younger mTBI groups. Non-injured control groups varied in rate of change of TL over time when compared to each mTBI group. TL of the four groups increased over time (*F*_(1, 27)_ = 5.837, *p* = 0.025); however, there were not significant differences between groups (*F*_(3, 27)_ = 2.412, *p* = 0.082).

### Symptom severity by group

**FIG. 2.**
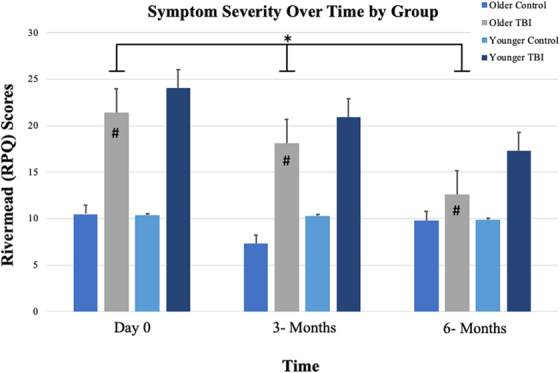
Significant aging-related differences were found between the older and younger mild TBI groups × time. The older mild TBI group had a significant change in symptom severity scores over time compared to younger mild TBI and non-injured control groups (*p* < 0.05). Line graph displays means ± standard error of the mean. *Indicates a significant main effect of time. ^#^Indicates a significant main effect of group (only in the older TBI group). TBI, traumatic brain injury.

We also evaluated comparisons between mTBI participants and their non-injured controls across three time points (day 0, 3 months, and 6 months) in symptom severity, as measured by total RPQ score (Fig. 2) and cognition, emotion, and somatic subscales. RM-ANOVA demonstrated significant aging-related differences within and between the older and younger mTBI groups by time. Mauchly's test of sphericity and Greenhouse-Geisser correction was used for within-group findings (*F*_(1.28, 34.5)_ = 5.77, *p* = 0.016). *Post hoc* comparisons using the SNK indicated that the older mTBI group was the only group that experienced significant change in symptom severity total scores over time. We did not observe significant differences in symptom severity total scores among the three other groups over time (*F*_(1, 27)_ = 2.86, *p* = 0.079).

We did not observe differences within and between groups by time on the RPQ cognition symptom subscale (*F*_(1.45, 39.1)_ = 0.058, *p* = 0.617; *F*_(4.35, 39.13)_ = 2.38, *p* = 0.077) by RM-ANOVA. There was a significant effect of time, but not group, on the RPQ emotion symptom subscale (*F*_(1.37, 37.1)_ = 4.18, *p* = 0.009) and on the RPQ somatic symptom subscale (*F*_(1.45, 39.2)_ = 10.4, *p* = 0.019). Mean emotion and somatic symptom subscale scores significantly decreased over time for baseline, 3 months, and at 6 months (Table 2).

**Table 2.** Symptom Severity by Subscale

Symptom subscale	Baseline (T1)	3 months (T1–T2)	6 months (T2–T3)	p value
Cognition	3.30 ± 0.51	3.20 ± 0.47	2.90 ± 0.71	0.077
Emotion	4.10 ± 0.67	3.10 ± 0.45	2.80 ± 0.61	0.009^*^
Somatic	8.60 ± 0.75	7.00 ± 0.76	5.80 ± 0.91	0.019^*^

Data are reported as means ± SEM and as means per symptom cluster/individual by subscale.

Key: T1: time 1 = baseline/day 0; T2: time 2 = 3 months; and T3: time 3 = 6 months.

^*^ Significant.

SEM, standard error of the mean.

### Symptom severity and telomere length

Shorter initial (day 0) TL was associated with higher symptom severity among the four groups at day 0 and predictive of symptom severity at 3 months, but not 6 months. Baseline and 3-month models explained ∼14% of the variance in the model, given that longer TL resulted in lower total symptom scores (*R*^2^ = 0.14, *F*_(1,29)_ = 4.87, *p* = 0.035; β = −7.7, *t*_(29)_ = −2.21, *p* = 0.035; and *R*^2^ = 0.14, *F*_(1,29)_ = 4.71, *p* = 0.038; β = −7.3, *t*(29) = −2.17, *p* = 0.038, respectively). Participants in the longer TL group had, on average, a score of 7.7 points lower on the RPQ at day 0 and a score of 7.3 points lower on the RPQ at 3 months. The 6-month model found no association between TL and symptom severity between groups (*R*^2^ = 0.03, *F*_(1,29)_ = 0.88, *p* = 0.357).

In addition, shorter TL (day 0) was associated with higher symptom severity based on the RPQ cognition subscale scores between groups at day 0 (baseline) and at 3 months, but not 6 months. The baseline model explained ∼22% of the variance, given that longer TL resulted in lower cognition subscale scores (*R*^2^ = 0.22, *F*_(1,29)_ = 8.21, *p* = 0.008; β = −3.19, *p* = 0.008). Participants in the longer TL group had a mean 3.2 points lower on the RPQ cognition subscale at day 0. The 3-month model explained ∼31% of the variance in cognitive symptoms, given that longer TLs were associated with lower cognition subscale scores (*R*^2^ = 0.31, *F*_(1,29)_ = 12.95, *p* = 0.001; β = −3.63, *p* = 0.001). Participants in the longer TL group had, on average, a 3.6-point reduction on the RPQ cognition subscale at 3 months. The 6-month model found no association between TL (day 0) and cognition subscale scores (*F*_(1,29)_ = 1.41, *p* = 0.245). We did not observe associations between TL (day 0) and the RPQ emotion and somatic subscales at day 0, 3 months, or 6 months (not reported).

## Discussion

In this pilot study, we examined TL because it may be useful to delineate the mechanistic underpinnings of post-TBI symptom severity in adults with mTBI. Our data suggest shorter TL associated with symptom severity (RPQ total and cognition subscale scores) among the groups at baseline and 3 months. We identified significant aging-related differences within mTBI groups over time in TL. Additionally, we found that adults who experienced mTBI had significant change in symptom severity scores over time. Thought to be passive players in cellular replication, recent research has emphasized telomeres as having a more active role in the promotion of cellular growth and survival.^8,9^ After TBI, the resulting cellular processes damage DNA, including telomeres. Recent literature also suggests that telomere shortening may be accelerated by TBI.^17,28^

Our results demonstrated that significant aging-related differences in TL were found within mTBI groups over time (day 0, 3 months, and 6 months; *p* = 0.025). Older adults with mTBI had shorter TL and experienced higher total symptom severity scores over time (day 0, 3 months, and 6 months; *p* = 0.016). These findings suggest that older adults with mTBI had significant worsening of changes in total symptom burden over time. Shorter TL at baseline was associated with higher total symptom burden between groups at baseline (*p* = 0.035) and predictive of symptom severity at 3 months (*p* = 0.038). When evaluating RPQ subscales, shorter TLs were associated with higher cognition scores and symptom burden between roups at day 0 (*p* = 0.008) and at 3 months (*p* = 0.008). Other studies in aging adults have demonstrated an association between higher levels of cognitive function (e.g., executive functioning, language, and memory) and longer TL.^29,30^ TBI groups are where we would expect a change in symptoms over time.

Although studies in otherwise healthy older adults have reported associations between TL and cognitive function, inconsistent findings occur across patient populations. In older trauma patients, shorter TLs were associated with poorer outcomes (e.g., lower likelihood of being discharged home) compared to younger trauma patients.^31^ In patients with breast cancer, cognitive impairment was associated with shorter TL and worsening cognitive symptoms (e.g., confusion, forgetfulness, and delusions).^32^ However, in Alzheimer's patients, a faster decline in cognition (i.e., executive functioning) was associated with longer TL.^33^

Our results demonstrated changes in TL among older adults with mTBI compared to non-injured controls across time. Shorter TLs have been reported in the saliva and peripheral skin cells (ear notch) of patients with TBI or in animal models of TBI. Similar to our findings, in a rodent fluid percussion injury model of TBI, middle-aged rats with TBI had worse motor deficits (e.g., more slips and falls on the beam task and less activity in the open field and elevated maze) and shorter TL in comparison to young adult rats post-TBI.^34^ Other researchers found telomere shortening to be associated with worse neurological outcomes in rats given repeated mTBIs. In a repeated mTBI (rmTBI) rat model, TL shortening was associated with rmTBI, and the rmTBI-induced changes in TL were correlated with diffusion-weighted magnetic resonance imaging changes.^35^ In another rodent model of rmTBI, Eyolfson and colleagues found that rmTBI was associated with reduced TL and functional changes (e.g., motor deficits, reduced aggression, and anxiety- and depressive-like behaviors).^36^ Our findings add to the clinical literature and support a role for TL in mTBI sequela in humans, adding new information on aging-related responses.

Chronic diseases (e.g., TBI) and the aging process are associated with shorter TL, but interventions (e.g., physical activity and diet) have been reported as protective of TL. Diets high in dietary fiber and unsaturated fats have been linked to longer TL,^37,38^ whereas high consumption of sugar and saturated fats has been associated with shortening of TL.^39,40^ Those effects could be mediated by oxidative stress and inflammation, given that antioxidant and -inflammatory properties of nutrients are associated with longer TL.^41,42^ The beneficial effects of physical activity on telomeres are associated with an increase in telomerase activity after an acute period of exercise.^43^ Physical activity may play a protective role on telomeres, but studies are warranted to establish the optimal exercise regime. Future studies are needed to investigate early interventions as other possible mechanisms contributing to the protective effects on TL and the mechanistic underpinnings of post-TBI symptom burden.

Several limitations to the study should be noted. A larger, more diverse patient sample (e.g., racial, ethnic, sexual, and gender minorities) is needed for the variability observed in adults with mTBI and to uncover associations with long-term symptoms. Current animal model methods collect ear notch samples for TL analyses; plasma, serum, and/or saliva specimens may provide further information about changes occurring in mTBI patients and should be explored in future studies.

## Conclusion

In conclusion, significant aging-related differences in TL were found in persons after mTBI. Older adults with mTBI were the only group that experienced significant change in symptom severity scores over time to 6 months. Shorter TLs were associated with higher post-injury symptom burden to 3 months in older and younger persons with mTBI. Large-scale, longitudinal studies of TL may be useful in predicting symptom severity over time in adults with mTBI.

## Abbreviations Used

CDC: Centers for Disease Control and Prevention
CT: computed tomography
FCS: fetal calf serum
mTBI: mild TBI
PBMC: peripheral blood mononuclear cell
PBS: phosphate-buffered saline
RM-ANOVA: repeated-measures analysis of variance
rmTBI: repeated mild TBI
RPQ: Rivermead Post-Concussion Symptoms Questionnaire
SCR: single-copy reference
SNK: Student Newman-Keuls
TBI: traumatic brain injury
TL: telomere length
UW: University of Washington
